# Human Papillomavirus Self-Sampling Attitudes Amongst Women Living with HIV Prior to a Self-Sampling Intervention

**DOI:** 10.3390/cancers18010014

**Published:** 2025-12-19

**Authors:** Sofia Nicolls, Emma Karlsen, Isabelle Boucoiran, Shariq Haider, Valérie Martel-Laferrière, Vanessa Poliquin, Marie-Louise Vachon, Sharon Walmsley, Alexander Wong, Sean Yaphe, Mark H. Yudin, Gina Ogilvie, Deborah Money, Elisabeth McClymont

**Affiliations:** 1 Department of Obstetrics & Gynaecology, University of British Columbia, Vancouver, BC V6H 3V4, Canada; 2 Women’s Health Research Institute, Vancouver, BC V5Z 1M9, Canada; 3 Départment de Microbiologie Infectiologie et d’Immunologie, Université de Montréal, Montréal, QC H3T 1J4, Canada; 4 Centre Hospitalier Universitaire Sainte-Justine, Montréal, QC H3T 1C5, Canada; 5 Department of Medicine, McMaster University, Hamilton, ON L8S 4K1, Canada; 6 McMaster University Hospital, Hamilton, ON L8S 4K1, Canada; 7 Centre de Recherche du Centre Hospitalier de l’Université de Montréal (CHUM), Montréal, QC H2X 0A9, Canada; 8 Department of Obstetrics, Gynaecology, and Reproductive Sciences, University of Manitoba, Winnipeg, MB R3T 2N2, Canada; 9 Health Sciences Centre, Winnipeg, MB R3A 1R9, Canada; 10 Départment de Microbiologie, Infectiologie, et d’Immunologie, Université Laval, Québec City, QC G1V 0A6, Canada; 11 Centre Hospitalier de l’Université Laval, Québec City, QC G1V 4G2, Canada; 12 Department of Medicine, University of Toronto, Toronto, ON M5S 1A8, Canada; 13 University Health Network, Toronto, ON M5G 2C4, Canada; 14 Department of Medicine, University of Saskatchewan, Saskatoon, SK S7N 5E5, Canada; 15 Regina Qu’Appelle Health Region, Regina, SK S4P 4W5, Canada; 16 Department of Family Medicine, McGill University, Montréal, QC H3A 1A1, Canada; 17 Centre Universitaire de Santé McGill, Montréal, QC H4A 3J1, Canada; 18 Department of Obstetrics and Gynaecology, University of Toronto, Toronto, ON M5S 1A8, Canada; 19 St. Michael’s Hospital, Toronto, ON M5B 1W8, Canada; 20 BC Cancer Research Institute, Vancouver, BC V5Z 1L3, Canada; 21 School of Population and Public Health, University of British Columbia, Vancouver, BC V6T 1Z3, Canada

**Keywords:** HIV, HPV, HPV self-sampling, cervical screening, acceptability, women

## Abstract

**Background/Objectives**: Our objective was to determine the acceptability of and attitudes towards HPV self-sampling among women with HIV and investigate any associations between self-sampling attitudes and participant demographic and clinical characteristics. **Methods:** Women with HIV aged 18–45 were given a description of HPV self-sampling and instructions on how to self-collect the sample. Participants completed a questionnaire assessing their perceptions of the acceptability and comfort of HPV self-sampling before using the self-sampling methodology. Responses were based on a 5-point Likert scale (strongly agree to strongly disagree) for each statement. Participants’ characteristics were included in bivariate analysis. Chi-square and Fisher’s exact tests were used to assess associations between questionnaire results and participant characteristics. **Results**: Of the 117 completed questionnaires, 79.6% of participants had a CD4+ T cell count ≥ 500 cells/mm^3^. Participants’ median age was 39 (IQR 34–43). One hundred participants (85.5%) felt confident they could collect their samples correctly, and 77.8% did not think they would experience difficulties with self-collection. Most participants (68.4%) preferred to self-collect their sample instead of provider-collected sampling. Ninety-six participants (82.1%) agreed they would likely use self-collection methods for future cervical screening. Many participants were concerned about receiving a positive HPV result (68.4%), passing HPV on to their partner(s) (75.7%), and disclosing their HPV status to friends/family (49.6%). **Conclusions**: Women with HIV seem to be accepting of HPV self-sampling as a cervical cancer screening methodology; however, many participants were concerned about the implications associated with a positive HPV test result.

## Simple Summary

Human Papillomavirus (HPV) causes almost all cervical cancers. Women with HIV experience higher rates of HPV infection that progresses more quickly to cervical cancer compared to women without HIV. Women with HIV are also less likely to participate in routine cervical cancer screening. Higher rates of violence and discrimination, as well as the stigma of an HIV diagnosis, have been reported as barriers to accessing cervical cancer screening within this population. HPV tests detect HPV DNA in the vagina and can be self-collected. The aim of our study was to determine the initial acceptability of and attitudes towards HPV self-sampling among a cohort of women with HIV in Canada. Among a sample of 117 women with HIV aged 18–45, most participants were accepting of HPV self-sampling and agreed that they would use self-sampling in the future; however, some participants were concerned about the implications of receiving a positive HPV result.

## 1. Introduction

In 2018, the World Health Organization (WHO) issued a call to action to eliminate cervical cancer by 2030 [1,2,3], making increased cervical cancer screening participation a top priority. Many well-established cervical cancer screening programs have begun to transition to primary Human Papillomavirus (HPV) testing due to the increased sensitivity of the test in detecting high-grade cervical intraepithelial neoplasia (CIN2+) compared to cervical cytology [4,5]. HPV tests can also be self-collected, which mitigates many barriers to testing and has the capacity to significantly increase routine cervical screening participation.

Cisgender women and persons with a cervix living with HIV (women with HIV) are especially vulnerable to HPV infection [6]. They are six times more likely to develop cervical cancer compared to women without HIV [1], are 36–44% less likely to clear oncogenic HPV infections compared to immunocompetent individuals, and experience higher rates of low and high-grade squamous intraepithelial lesions (LSIL and HSIL, respectively) [7].

Despite women with HIV’s increased susceptibility to persistent oncogenic HPV infections, rapid progression to precancerous lesions, and cervical cancer, they are less likely to participate in cervical screening programs than women without HIV (58% vs. 81%) [3,8]. Women with HIV often face barriers to accessing care, such as higher rates of gender-based violence, discrimination, and the stigma of an HIV or HPV diagnosis [8,9,10,11]. Previous experiences of HIV stigma and fear of HIV status disclosure in healthcare settings prevent many women with HIV from interacting with sites of clinical care [12]. Women with HIV who are immigrants, have lower socioeconomic status, and are from racialized communities often experience intersecting barriers to accessing and navigating cervical cancer screening programs [8,9,10,11]. Due to these disparities, women with HIV may have different attitudes towards cervical cancer screening compared to the general population.

Certain provincial jurisdictions in Canada have implemented HPV testing with collection via self-sampling as the primary method of screening for cervical cancer, whereby patients can opt to collect their own HPV test and mail their sample for laboratory testing [10,13]. Although previous research has determined that HPV self-sampling increases cervical cancer screening participation and is an accepted screening method for individuals without HIV [14,15], there are currently no data on women with HIV’s attitudes and perceptions of HPV self-sampling in Canada. Data from other global regions suggest that HPV self-sampling may be acceptable to women with HIV in those settings [16,17]. HPV self-sampling may have significant clinical implications in reducing barriers to testing for women with HIV and, ultimately, preventing cervical cancer within this population. We anticipate that women with HIV will have higher acceptability and preferences towards self-collection compared to women without HIV, particularly compared to individuals who are highly engaged in routine cervical screening. Being able to self-collect the HPV sample may provide women with HIV with the agency to decide which spaces may be safe for them to self-collect, mitigating geographical barriers (having to travel far distances to access health clinics) and socio-cultural barriers (judgment, HIV stigma, and discrimination, which can be further exasperated by racism, sexism, and classism) [11]. As HPV self-sampling becomes more commonly utilized globally [14,15,18,19], it is vital to investigate women with HIV’s attitudes and perceptions of this screening tool.

The primary objective of this research is to determine the acceptability of HPV self-sampling among women with HIV. The secondary objective was to assess the attitudes towards self-sampling for HPV among women living with HIV. Additional exploratory objectives were to investigate any associations between self-sampling acceptability and participant demographic/clinical characteristics. Our findings may provide opportunities for community-based education and the development of specific screening resources for women with HIV.

## 2. Materials and Methods

### 2.1. Participant Recruitment

Participants were recruited for the NOVA-HIV Study, a randomized clinical trial assessing the efficacy of reduced dosing for the nonavalent HPV vaccine in women with HIV. Participants were included in the randomized trial if they were a woman and/or person with a cervix with a confirmed diagnosis of HIV, were not pregnant and not trying to become pregnant, and were within the specified study age group (18–45 years) at the time of enrollment. Participants were not enrolled in the study if they were allergic to the vaccine or any of its components or had previously received an HPV vaccine.

All participants completed an HPV self-sampling attitudes questionnaire during their first study visit. Participants were given a brief description of HPV self-sampling and instructions on collecting their sample. Participant attitude questionnaires were completed before participants self-collected to evaluate their initial perceptions of HPV self-sampling. Participant demographic and clinical characteristics (gender, ethnicity, HIV diagnosis date, HIV viral load, CD4+ T-cell count, and antiretroviral medication use) were documented during the first study visit. Information regarding cervical cytology history (date of last Pap/HPV test, Pap/HPV test results, etc.) was abstracted from patient charts. Ethics approval was granted at the study coordinating centre by the University of British Columbia Children’s and Women’s Research Ethics Board (H22-02639), and at all participating sites. All participants gave written informed consent.

### 2.2. HPV Self-Sampling Attitudes Questionnaire

HPV self-sampling questionnaires were completed at study sites using REDCap (Research Electronic Data Capture). The HPV self-sampling attitudes questionnaire consists of 22 items (Appendix A). The questionnaire was adapted from the surveys used in the CervixCheck and FOCAL studies [20,21]. The prior surveys were pilot-tested for clarity and comprehension of questions. Questionnaire responses were based on a 5-point Likert scale (strongly agree to strongly disagree, very likely to very unlikely, or very concerned to not at all concerned, depending on the wording and structure of the questionnaire prompt). If participants chose “strongly agree” or “agree” to a particular statement, participants’ responses were categorized as “agree”. Similarly, if participants chose “strongly disagree” or “disagree”, their responses were categorized as “disagree”.

### 2.3. Outcomes

The primary outcome of interest was to determine participants’ acceptance of HPV self-sampling. Self-sampling acceptability was based on participants’ responses to the prompt, “If you had the opportunity, how likely are you to use self-collection in the future for cervical cancer screening?” If participants chose “likely” or “very likely” in response to the prompt, then they were considered “accepting” of HPV self-sampling. If participants chose “unsure”, “unlikely”, or “very unlikely”, they were considered “not accepting” of HPV self-sampling. Participants’ cervical screening preferences were analyzed based on the question, “Assuming that both HPV self-collection and having a healthcare provider collect a cervical sample are equally safe and effective for testing, what would you prefer in a future screening program?” Potential answers to this question are included in Appendix A. The secondary outcomes of interest were to understand participants’ trust in the HPV self-sampling methodology (compared to Pap testing), perceived ease of sample collection, and concerns surrounding their overall health if they were to receive a positive HPV self-sampling result.

### 2.4. Statistical Analysis

Likert scales were summarized as “agree”, “disagree”, or “unsure” for each statement (except for the acceptability prompt, which considered “unsure” respondents to be “not accepting” of HPV self-sampling). Demographic and clinical variables were included in a bivariate analysis. Chi-square and Fisher’s exact tests were used to assess relationships between questionnaire results and categorical clinical characteristics using unadjusted prevalence ratios (PRs). All statistical analysis was performed using R (version 1.1.4) [22].

## 3. Results

### 3.1. Study Population

Participants aged 18–45 were recruited from 11 study sites across five provinces in Canada as part of a reduced-dosing clinical trial of the nonavalent HPV vaccine. For this preliminary analysis, we collected questionnaires from the first 117 participants who enrolled in the study. At the time of recruitment, participants were residing in British Columbia (47.0%), Saskatchewan (3.4%), Ontario (27.3%), and Québec (22.2%) (Table 1). Participants’ median age was 39 ([IQR]: 34–43, range: 19–45). Participants’ ethnicity was predominantly African, Caribbean, or Black (59.0%) or White (21.4%). When asked about their gender identity, all participants identified as cisgender women. One hundred and thirteen (97.4%) participants were taking antiretroviral medications, and 90.2% were virologically suppressed (<50 copies/mL). Ninety participants (79.6%) had a CD4+ T cell count greater than 500 copies/mm^3^.

### 3.2. Cervical Cytology

In Canada, the youngest age to start screening for cervical cancer using an HPV test is 25 years. For women with HIV, cervical screening is recommended every one or three years via Pap or HPV testing, respectively, depending on the standard screening methodology of the region. Of participants who were sexually active and ≥25 years of age (n = 112), 7.5% had previously received a positive HPV test result, and 25.7% had previously received an abnormal Pap or HPV result. Among participants who had received an abnormal Pap result and had available cytology data (n = 22), the highest cytology result they received was ASCUS in 36.4%, CIN1/LSIL in 36.4%, and CIN2/3 or HSIL in 27.2% of participants. Sixteen participants (17.0%) had received a Pap/HPV test between one and three years before the start of the study, and 9.6% of participants had not received a Pap test in the last three years (n = 9) (Table 1).

### 3.3. HPV Self-Sampling Attitudes

**Figure 1:**
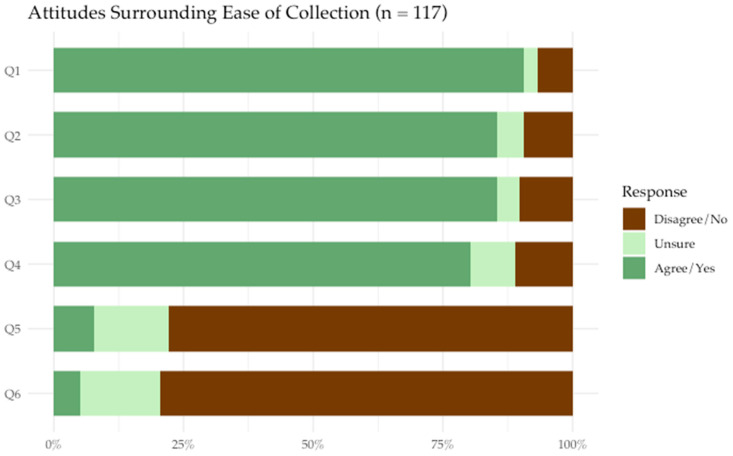
Participants’ responses to the following prompts regarding ease of collection (n = 117).

**Figure 2:**
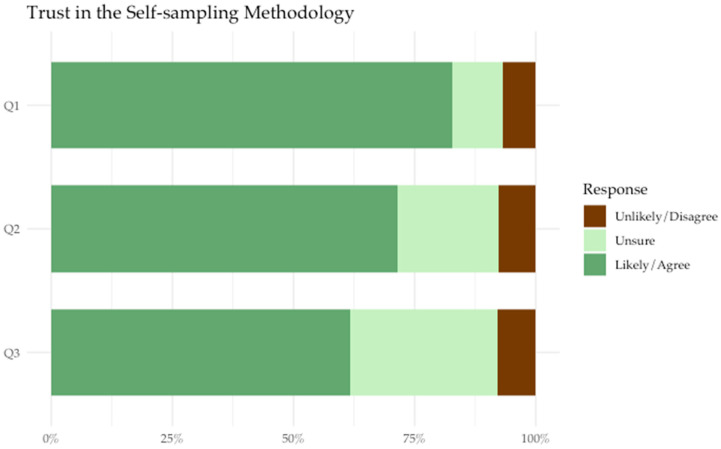
Participants’ responses to the following prompts regarding trust in the self-sampling methodology (n = 117).

The majority of participants were accepting of HPV self-sampling as a method of screening for cervical cancer. Participants felt that they were likely to use HPV self-sampling in the future (82.1%) and would recommend HPV self-sampling to other women they knew (82.8%). Most agreed that HPV self-sampling instructions seemed easy to follow (90.6%), felt confident that they could collect the sample correctly (85.5%), and did not think that self-collection would be painful (79.5%) (Figure 1). Compared to previous screening methodologies, 61.7% of participants agreed that they trusted HPV self-sampling to be as accurate as Pap testing, and 71.6% of participants agreed that HPV screening was as safe as Pap testing (Figure 2). We did not identify any statistically significant associations between participant demographic/clinical characteristics and HPV self-sampling acceptance in our analysis (Table 1).

In response to the following question, “Assuming that both HPV self-collection and having a healthcare provider collect a cervical sample are equally safe and effective for testing, what would you prefer in a future screening program?”, 68.4% of participants chose HPV self-sampling as their preferred method of screening, 19.7% preferred a provider-collected sample, and 10.3% of participants reported not having a screening method preference. For those who disagreed that they were likely to use self-collection in the future (n = 13), participants felt that they wouldn’t like taking their own test (61.5%), that they didn’t think that the self-collected screening test worked (7.7%), and were scared of what the test results might show (15.4%) (Appendix A).

**Figure 3:**
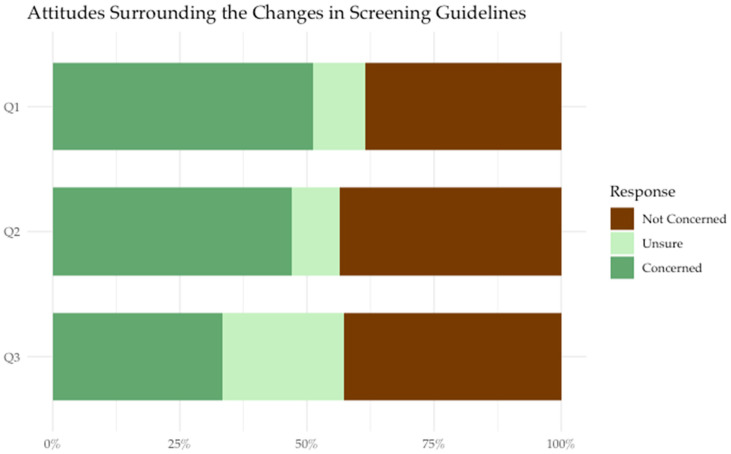
Participants’ responses to the following prompts about changes in cancer screening guidelines (n = 117).

**Figure 4:**
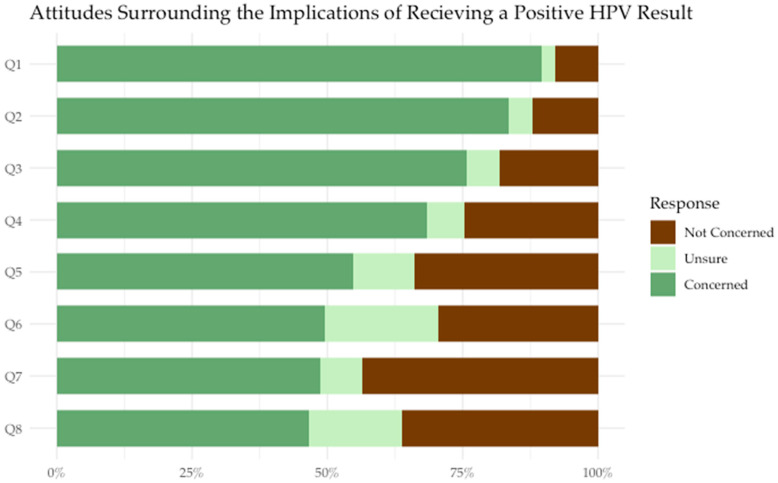
Participants’ responses to the following prompts regarding the concern of testing positive for HPV (n = 117).

Most participants expressed concern about the impact that receiving a positive HPV self-sampling result would have on their overall health. Some participants expressed concerns about potential changes that may be associated with the implementation of HPV screening, including the longer length of time between screens (every 3 years after receiving a negative HPV test result) (33.3% concerned; 23.9% unsure), and not seeing a healthcare provider for a Pap test (47% concerned, 9.4% unsure) (Figure 3). Participants also expressed concern surrounding the follow-up recommended after a positive HPV result (51.3% concerned, 10.3% unsure), as well as the social and interpersonal implications of testing positive, such as knowing when they got HPV (54.8% concerned, 11.3% unsure) or knowing who gave them HPV (46.1% concerned, 17.2% unsure). Participants also reported concern and uncertainty about disclosing their HPV status to their partner (48.7% concerned, 7.8% unsure) or friends and family (49.6% concerned, 20.9% unsure). Finally, participants expressed concern about passing HPV on to their partner(s) (75.7% concerned, 6.1% unsure) (Figure 4).

Q1: The instructions above for collecting a swab would be easy to follow.

Q2: Self-collecting a vaginal sample would be easy to do.

Q3: I feel confident that I would be able to collect the sample correctly

Q4: The self-collection swab (which is like a long Q-tip) would be comfortable to use.

Q5: Do you think you would experience any other difficulties with self-collection?

Q6: Do you think self-collection would cause you any pain or discomfort that would discourage you from self-collecting?

Q1: Does self-collection HPV screening sound like something you would be likely to recommend to other women you know? (Prefer not to answer = 1)

Q2: I trust that HPV self-screening is as safe as Pap testing. (Missing = 2)

Q3: I trust that HPV self-screening is as accurate as Pap testing. (Missing = 1)

Q1: The follow-up recommended after a positive HPV result.

Q2: Not seeing a healthcare provider for a Pap test.

Q3: The longer length of time between screens (5 years).

Q1: Developing cervical cancer in the next 5 years (Not applicable = 1, Prefer not to answer = 1).

Q2: My overall general health (Not applicable = 1, Prefer not to answer = 1).

Q3: Passing HPV on to my partner (Not applicable = 1, Prefer not to answer = 1).

Q4: Receiving an HPV positive result.

Q5: Knowing when I got HPV (Not applicable = 1, Prefer not to answer = 1).

Q6: Telling friends or family (Not applicable = 1, Prefer not to answer = 1).

Q7: Telling my partner (Not applicable = 1, Prefer not to answer = 1).

Q8: Knowing who gave me HPV (Not applicable = 1).

## 4. Discussion

### 4.1. Main Findings

This study showed that self-sampling was an acceptable method of screening for cervical cancer (82.1% found it to be acceptable). The attitudes to self-sampling were that it was considered a preferred method of future HPV testing (compared to provider-collected swabs) and they indicated that HPV self-sampling would be easy and safe to use, and more than half of participants felt that HPV self-sampling was as accurate (61.7%) and as safe (71.6%) as Pap testing although concerns were expressed around the implications of an HPV diagnosis. Demographic/clinical characteristics of the participants did not differ by acceptance of HPV self-sampling.

Our findings are similar to a self-sampling acceptability study among women with HIV in the United States, where 90% of participants agreed that self-sampling was convenient and comfortable [23], and 85% of the participants would recommend self-sampling to family and friends. The observed rates of acceptability within our cohort of women with HIV are higher than what has been previously reported in individuals without HIV in Canada. In a study assessing HPV self-sampling attitudes among women, only 52.1% of respondents reported willingness to self-collect [24]. A potential explanation for this difference in acceptability could be that the cohort of women without HIV was well-engaged in cervical cancer screening, and therefore may be less willing to change cervical screening methods (from provider-collected samples to self-collection). Additionally, many women with HIV report negative, stigmatizing experiences accessing care, and may be more accepting of screening methods that enable them to take care into their own hands [11].

### 4.2. Changes in Continuity of Care

Although the majority of participants found HPV self-sampling to be an acceptable method of cervical cancer screening, 47% of participants expressed concern about not seeing a healthcare provider for a Pap test, and 33% were concerned about the changes in duration between screens associated with an HPV-based screening program (screening every three years upon receiving a negative HPV test compared to every year for Pap tests, according to the British Columbian HPV testing guidelines) [13]. These concerns may be due to a lack of information about why it is safe to extend cervical screening intervals when using HPV self-sampling. Without appropriate guidance and education, women may perceive these guideline changes as a decrease in their quality of care. It is important for healthcare providers to explain to their patients the rationale behind these changes and how self-sampling improves their care as a superior test and provides them with more choices regarding the method of cervical cancer screening.

### 4.3. Trust in the Safety and Effectiveness of HPV Self-Sampling

Although more than 50% of participants trusted that HPV self-testing was as accurate and safe as provider-collected Pap smears, there was a significant proportion of participants who disagreed with, or expressed uncertainty in response to, these questionnaire prompts. Participants may be concerned about the accuracy of HPV testing (compared to Pap testing) and the sensitivity of self-collected (versus provider-collected) HPV samples. Although the medical and scientific community is aware of the extensive scientific literature showing the improved sensitivity of HPV testing compared to liquid-based cytology [4,5], and the comparable accuracy of self-collected (versus provider-collected) vaginal samples [25], this knowledge must be appropriately disseminated to the public, especially among populations that are at higher risk of developing cervical cancer.

### 4.4. Interpersonal Implications of Testing Positive for HPV

Many participants also expressed concern regarding the implications of a positive HPV test, including disclosure of their HPV status to partner(s), friends, and family. This may allude to a shift in societal attitudes surrounding cervical cancer screening in Canada. Since HPV self-sampling tests for viral nucleic acid in the vagina (instead of dysplastic cells on the cervix), perspectives may shift focus from cervical cancer prevention to viewing HPV self-sampling as testing for a sexually transmitted infection (STI). Participants expressed concern about how they got HPV, who gave them HPV, and transmitting HPV to their partner(s). These attitudes have been largely absent in conversations around Pap smears. Questionnaire responses suggest that participants may be concerned about the perceived stigma associated with a positive HPV test (i.e., fear of discrimination and/or negative attitudes of family members or partners towards testing positive for HPV). Although similar concerns have been reported in populations without HIV, this may be particularly concerning for populations with HIV, as many women with HIV have previous negative experiences with STI testing and HIV disclosure [12]. HPV-related stigma has the capacity to prevent women with HIV from accessing cervical cancer screening, as the perceived stigma surrounding STIs contributes to decreased participation in STI testing and care [26].

### 4.5. Clinical Implications

In Canada, screening methodologies and cervical cancer prevention programs differ between provincial jurisdictions. Although HPV testing has been implemented in select provinces, we found no significant statistical association between the acceptability of HPV self-sampling and participants’ province of residence. For example, participants who reside in BC, where HPV testing and self-sampling have been implemented for almost two years, did not have different responses in self-sampling acceptability compared to participants from provinces that still use Pap tests (such as Saskatchewan) or who use HPV tests but have not yet implemented self-sampling (such as Ontario or Québec). As jurisdictions begin to implement HPV testing and self-sampling into their cervical screening programs, it is essential to provide education to women with HIV on the new changes surrounding cervical cancer screening.

### 4.6. Strengths and Limitations

There were many strengths to this paper. There has been very limited data investigating the self-sampling attitudes among women with HIV in Canada, which this study now provides herein. This multi-site research study recruited participants across Canada, providing a valuable comparison of perspectives between women with HIV in provinces with different cervical cancer screening programs. There are, however, limitations within our study. Firstly, we did not collect participants’ socioeconomic status, which has been previously associated with their willingness to self-collect an HPV sample [24]. Another limitation of our study is that we did not provide measures to assess levels of knowledge of HPV and cervical cancer prevention methods within our study design. Previous research has shown that a lack of knowledge about HPV self-sampling is associated with decreased acceptance, uptake of HPV testing, and participation in cervical cancer screening programs [27]. However, basic information about HPV testing was provided prior to completion of the questionnaire. It is also important to note that our cohort of women with HIV was recruited from clinics for HIV and gynecologic care located in urban cities, and thus, we may not have adequately captured the HPV self-sampling attitudes of women with HIV who live in rural parts of Canada. Women with HIV who reside in rural and remote areas of Canada often disproportionately experience barriers to accessing care, including long travel distances to attend medical appointments and low numbers of HIV-specialist physicians per capita [28,29,30,31]. Additionally, lower rates of HIV-related knowledge are observed in remote regions of Canada, which often contributes to higher rates of HIV-related stigma in these areas [28,31]. Recruitment methods may also impact the generalizability of the results to women with HIV who are not engaged in care. This cohort of women with HIV attended the clinic and were willing to participate in a vaccine trial, and may have different views than women with HIV who have limited care. All the participants in our study identified as cisgender women, and thus, we were unable to investigate HPV self-sampling attitudes among trans and gender-diverse (TGD) individuals with a cervix, who experience additional difficulties accessing gynaecologic care (including cervical cancer screening) compared to cisgender women [32]. Future research examining HPV self-sampling attitudes among rural women with HIV and TGD individuals is needed, as HPV self-sampling may be a beneficial tool in dismantling cervical screening barriers within these populations.

## 5. Conclusions

Although HPV self-sampling was an acceptable screening method among our cohort of women with HIV, many participants expressed concern about the implications of receiving a positive HPV test result. A significant proportion (~30–40%) of women with HIV in our study felt that HPV self-sampling was less accurate and safe than Pap testing. HPV self-sampling has the tremendous capacity to increase access to routine cancer screening among women with HIV, and, as a result, prevent cervical cancer within this population. To maximize its potential, more efforts need to be made to increase education and knowledge about HPV testing and self-sampling. Collaboration with patient partners and community stakeholders in developing screening options that best fit the needs of women with HIV will be necessary to eliminate cervical cancer in Canada and to create equitable and empowering healthcare for women with HIV.

## Figures and Tables

**Table 1:** Demographic and clinical characteristics of participants who completed the HPV self-sampling questionnaire (n = 117).

Demographic and Clinical Characteristics	Total Number of Participants n = 117 (%)	If You Had the Opportunity, How Likely Are You to Use Self-Collection in the Future for Cervical Cancer Screening?			Unadjusted Prevalence Ratio (PR) (95% CI)		*p* Value
		Likely 96 (82.1)	Unlikely/Unsure 21 (17.9)				
**Age, median (IQR)**	39 (34–43)	39 (34–43)	39 (35–43)		n/a		0.892
**Race, n (%)**							
African/Caribbean/Black	69 (59.0)	56 (58.3)	13 (61.9)		Ref		
Hispanic/Latino/Latina	4 (3.4)	3 (3.1)	1 (4.8)		0.92 (0.52–1.65)		1
Indigenous from North America	8 (6.8)	7 (7.3)	1 (4.8)		1.08 (0.81–1.43)		1
South/East/Southeast Asian/Middle Eastern	11 (9.4)	9 (9.4)	2 (9.5)		1.0 (0.75–1.36)		1
White	25 (21.4)	21 (21.9)	4 (19.0)		1.0 (0.84- 1.27)		1
**Currently taking antiretroviral medication, n (%)**							
Yes	114 (97.4)	95 (99.0)	19 (90.5)		Ref		
No	3 (2.6)	1 (1.0)	2 (9.5)		4.0 (1.63–9.83)		0.083
**CD4+ T cell count, n (%)** (cells/µL)							
>500	90 (79.6)	76 (81.7)		14 (70.0)		Ref	
<500	23 (20.4)	17 (18.3)		6 (30.0)		1.68 (0.72–3.88)	0.24
Missing = 4		3		1			
**Baseline suppressed viral load, n (%)** (<50 copies/mL)							
Yes	101 (90.2)	84 (91.3)		17 (85.0)		Ref	
No	11 (9.8)	8 (8.7)		3 (15.0)		1.62 (0.56–4.67)	0.411
Missing = 5		4		1			
**Province of residence, n (%)**							
British Columbia	55 (47.0)	47 (49.0)		8 (38.1)		Ref	
Ontario	32 (27.3)	23 (23.9)		9 (42.9)		0.84 (0.66–1.07)	0.12
Québec	26 (22.2)	23 (23.9)		3 (14.3)		1.03 (0.87–1.23)	1
Saskatchewan	4 (3.4)	3 (3.1)		1 (4.8)		0.88 (0.49–1.56)	0.49
**Previous sexual intercourse, n (%)**							
Yes	113 (97.4)	93 (97.9)		20 (95.2)		Ref	
No	3 (2.6)	2 (2.1)		1 (4.8)		1.88 (0.36–9.80)	0.45
Missing = 1		1					
**Previous abnormal pap test or a positive HPV result, n (%)**							
Yes	28 (25.7)	23 (25.3)		5 (27.8)		0.98 (0.80–1.19)	0.824
No	81 (74.3)	68 (74.7)		13 (72.2)		Ref	
Missing = 3		1		2			
**History of a positive HPV test**							
Yes	8 (7.5)	6 (6.8)		2 (11.1)		0.90 (0.596–1.350)	0.621
No	98 (92.5)	82 (93.2)		16 (88.9)		Ref	
Missing = 6		4		2			
**Time since last Pap test/HPV test**							
<1 year	69 (73.4)	54 (70.1)		15 (88.2)		Ref	
1–3 years	16 (17.0)	15 (19.5)		1 (5.9)		1.20 (1.00–1.43)	0.285
>3 years	9 (9.6)	8 (10.4)		1 (5.9)		1.13 (0.87–1.48)	0.676
Missing = 18		15		3			
**Worst Pap/cytology result ever**							
No abnormal Pap results	81 (78.6)	68 (79.1)		13 (76.5)		Ref	
CINI or LSIL	8 (7.8)	6 (7.0)		2 (11.8)		0.89 (0.59–1.35)	0.62
CIN II/III or HSIL	6 (5.8)	6 (7.0)		0		0.89 (0.59–1.35)	0.62
Invasive Cervical Cancer	0	0		0		-	-
ASCUS	8 (7.8)	6 (7.0)		2 (11.8)		1.19 (1.08–1.31)	0.58
Missing = 6		5		1			

## Data Availability

Aggregate data presented in this study are available upon reasonable request from the corresponding author due to privacy and confidentiality requirements.

## Abbreviations

The following abbreviations are used in this manuscript:

WHO	World Health Organization
HPV	Human papillomavirus
CIN2+	High-grade cervical intraepithelial neoplasia
LSIL	Low-grade squamous intraepithelial lesions
HSIL	High-grade squamous intraepithelial lesions
PR	Prevalence ratio
IQR	Interquartile range
ASCUS	Atypical squamous cells of undetermined significance
STI	Sexually transmitted infections
TGD	Trans and gender-diverse

## Appendix A

**Table A1:** Participants’ attitudes about the safety and comfort of using HPV self-sampling methodologies.

Participant Attitudes Surrounding HPV Self-Sampling Safety/Comfort	Number of Participants n = 117 (%)
**The instructions above for collecting a swab would be easy to follow**	
Agree	106 (90.6)
Disagree	8 (6.8)
Unsure	3 (2.6)
**Self-collecting a vaginal sample would be easy to do**	
Agree	100 (85.5)
Disagree	11 (9.4)
Unsure	6 (5.1)
**I feel confident that I would be able to collect the sample correctly**	
Agree	100 (85.5)
Disagree	12 (10.3)
Unsure	5 (4.3)
**The self-collection swab (which is like a long Q-tip) would be comfortable to use**	
Agree	94 (80.3)
Disagree	13 (11.1)
Unsure	10 (8.5)
**Do you think self-collection would cause you any pain or discomfort that would discourage you from self-collecting?**	
Yes	6 (5.1)
No	93 (79.5)
Unsure	18 (15.4)
**Do you think you would experience any other difficulties with self-collection?**	
Yes	9 (7.7)
No	91 (77.8)
Unsure	17 (14.5)

**Table A2:** Participants’ responses to questions regarding their trust and confidence in the HPV self-sampling procedures.

Participants’ Trust in Utilizing HPV Self-Sampling Methodologies	Number of Participants (%) N = 117
**Does self-collection HPV screening sound like something you would be likely to recommend to other women you know?**	
Likely	96 (82.8)
Unlikely	8 (6.9)
Unsure	12 (10.3)
Prefer not to answer = 1	
**I trust that HPV self-screening is as accurate as Pap testing**	
Agree	71 (61.7)
Disagree	9 (7.8)
Unsure	35 (30.4)
Missing = 2	
**I trust that HPV self-screening is as safe as Pap testing**	
Agree	83 (71.6)
Disagree	9 (7.8)
Unsure	24 (20.7)
Missing = 1	
**Assuming that both HPV self-collection and having a healthcare provider collect a cervical sample are equally safe and effective for testing, what would you prefer in a future screening program?**	
Self-collection	80 (68.4)
Sample taken by a physician	23 (19.7)
I don’t intend to get cervical screening in the future	0
I have no preference	12 (10.3)
I don’t know	1 (0.8)
I prefer not to answer	1 (0.8)
**If you had the opportunity, how likely are you to use self-collection in the future for cervical cancer screening?**	
Likely	96 (82.1)
unlikely	13 (11.1)
Unsure	8 (6.8)
**If you answered “unlikely” to the above question, please select the main reason:**	
I wouldn’t like taking my own test	8 (61.5)
I don’t think I can safely take my own test	1 (7.7)
I don’t think the self-collected screening test works	1 (7.7)
I’m scared of what the results may show	2 (15.4)
Other	1 (7.7)

**Table A3:** Participants’ responses about their overall health and safety concerning the HPV self-sampling methodologies.

Participants’ Concern for Their Overall Health with Regard to HPV Self-Sampling Methodology	Number of Participants (%) N = 117
**The longer length of time between screens (every 5 years)**	
Not concerned	50 (42.7)
Concerned	39 (33.3)
Unsure	28 (23.9)
**Receiving an HPV-positive result**	
Not concerned	29 (24.8)
Concerned	80 (68.4)
Unsure	8 (6.8)
**The follow-up recommended after a positive HPV result**	
Not concerned	45 (38.5)
Concerned	60 (51.3)
Unsure	12 (10.2)
**Not seeing a healthcare provider for a Pap test**	
Not concerned	51 (43.6)
Concerned	55 (47.0)
Unsure	11 (9.4)
**Knowing who gave me HPV**	
Not concerned	42 (36.2)
Concerned	54 (46.6)
Unsure	20 (17.2)
Not applicable = 1	
**Knowing when I got HPV**	
Not concerned	39 (33.9)
Concerned	63 (54.8)
Unsure	13 (11.3)
Not applicable = 1	
Prefer not to answer = 1	
**Developing cervical cancer in the next 5 years**	
Not concerned	9 (7.8)
Concerned	103 (89.6)
Unsure	3 (2.6)
Not applicable = 1	
Prefer not to answer = 1	
**Telling my partner**	
Not concerned	50 (43.5)
Concerned	56 (48.7)
Unsure	9 (7.8)
Not applicable = 1	
Prefer not to answer = 1	
**Passing HPV on to my partner**	
Not concerned	21 (18.3)
Concerned	87 (75.7)
Unsure	7 (6.1)
Not applicable = 1	
Prefer not to answer = 1	
**Telling friends or family**	
Not concerned	34 (29.6)
Concerned	57 (49.6)
Unsure	24 (20.9)
Not applicable = 1	
Prefer not to answer = 1	
**My overall general health**	
Not concerned	14 (12.2)
Concerned	96 (83.5)
Unsure	5 (4.3)
Not applicable = 1	
Prefer not to answer = 1	
